# Supplementary material for: Modelling the synergistic effect of bacteriophage and antibiotics on bacteria: Killers and drivers of resistance evolution
Source: PLoS Comput Biol. 2022 Nov 30;18(11):e1010746. doi: 10.1371/journal.pcbi.1010746 (PMC9744316; doi:10.1371/journal.pcbi.1010746)
Supplement: Attachment — Submitted filename: review.docx [file pcbi.1010746.s008.docx]

The paper titled “Modelling the synergistic effect of bacteriophage and antibiotics on bacteria: killers and drivers of resistance evolution” explores how phage capable of generalized transduction impacts the efficacy of phage-antibiotics combination therapy. The authors claim that phage and antibiotics can work in combination to kill the bacterial population or to generate multidrug-resistant strains, the outcome will depend on the concentration and timing of phage and antibiotics application. The authors support their claims using a previously published mathematical model [5] which they extend by adding the pharmacodynamics of erythromycin and tetracycline, parameterized with new *in vitro* data from this study.

The claims of this manuscript contribute to the nascent field of phage-antibiotics combination therapy, proposing that generalized transduction and the timing and concentration of phage and antibiotics application shape the outcome of the combined treatment. However, these claims are not novel as there are previous experimental papers proposing similar ideas on the timing and concentration of phage-antibiotics application. For example, some studies have shown that pre-exposing biofilms to phage before adding the antibiotics produce the maximum anti-biofilm activity [1-3] while pre-exposing biofilms to antibiotics before adding phage can have antagonistic effects [2]. Similarly, the effects of timing and concentration of the combined treatment have been explored in planktonic bacteria, treating a bacterial population with phage prior to the addition of antibiotics resulted in the greatest bacterial density reduction [4].

I have outlined some major and minor comments below so the authors can consider them to improve their manuscript and to provide further support to their main claims.

**Major comments**

1. Provide explanation/justification for some key aspects of the mathematical model. The main claims of the manuscript about the effects of timing and concentration and generalized transduction on the combination therapy outcome are supported by the mathematical model described in the manuscript. However, the model lacks some justification in some of its key aspects. For example, in equation 3 there is no explanation why the antibiotic effect (ε_i,j_) is coupled to the maximum bacterial growth rate (μmax_j_), as if an increase in the maximum bacterial growth rate increases the antibiotic effect. Then, equations 4-6 describe the population dynamics of the different bacterial strains but there is no explanation why their growth terms are coupled to the phage infection. For example, the growth term of bacteria resistant to erythromycin (B_E_) is described as $\mu_{E}(B_{E}- ((\varphi_{L}+{\varphi_{T})B_{E}))}$but there is no justification why phage infection negatively impacts replication of $B_{E}$. Then, is not clear how the parameters for bacterial growth and phage predations were obtained, whether they were calculated using the *in vitro* data from this manuscript or if they were obtained from [5]. Explaining key information that is missed from the components described above can be helpful and provide further support to the main results of the manuscript.
2. Proposing *in vitro* experiments to further support modeling outcomes. I think the manuscript could benefit from incorporating some simple experimental assays. The authors have the needed bacterial and phage strains for the proposed *in vitro* experiments. I propose to do some liquid co-cultures of the two bacterial strains resistant to single antibiotics and add phage and the antibiotics sequentially or simultaneously. The main idea is to quantify the effects of the combined treatment over time (via optical density or CFU counts) such that the experiment last ~24 to 48h. For example, in one experimental condition phage can be added first and later the antibiotics while in another condition antibiotics are added first and then the phage. In a third *in vitro* experiment phage and antibiotics are added simultaneously. Different concentrations of phage and antibiotics can be tested but I consider that using two is enough (high and low concentrations for both phage and antibiotics). The phage being capable of generalized transduction will produce the multidrug-resistant strains during the time frame of the suggested experiments (~24 to 48h); the multidrug-resistant strains could be selected when exposed to both antibiotics and then quantify. I consider that producing these experiments could provide further support to the modeling results. Even if the results from the *in vitro* experiments are different from the modeling outcomes, then the new information generated via experiments could be helpful to adjust the mathematical model.

**Minor comments**

1. Line 57: add cites after the sentence, “Some previous studies showed that phage and antibiotics work synergistically to clear bacteria [add cites here]”.
2. Table 1, what are the correct units for the carrying capacity, *Nmax*, bacteria (CFU) or CFU/ml?
3. Table 1, what is the correct reference for bacterial growth and phage parameters, article 22 or 23?
4. Table 1, what are the units for the half maximal effective concentration for the different antibiotics? mg/L?
5. In line 296-297 the authors mentioned that the MIC of erythromycin for the sensitive strain was 0.25 mg/L and the same was found for tetracycline. But in line 320 authors mentioned that they simulated the addition of 1 mg/L of antibiotic which is 4 x MIC for susceptible strains and not 2 x MIC as stated. So clarify, if the MIC for susceptible strains is 0.25 mg/L or 0.5 mg/L.
6. The error described above is found in different parts of the results secion, for example, see line 346 from the caption of figure 3.
7. I think it would be clearer to show population dynamics plots for some of the combination therapy scenarios described in lines 382-392 than the current figure 5a.
8. Line 476, maybe you could try to use parameters from the literature about antibiotic decay rates.
9. Lines 520-528, authors should point to the figures when mentioning the results described in those lines.
10. Lines 522-523, 2 mg/L of antibiotics is 4 or 8 x MIC?
11. Line 545, it should say Figure 6c instead of Figure 5c.
12. Lines 551-553, maybe clarify if both sequential treatments can still lead to bacterial elimination.
13. Lines 581-582 it would be interesting to explore scenario where phage resistant bacteria are allowed to emerge. How does phage resistance affect the generation of multidrug-resistant bacteria? Or could phage resistance drive combined treatment to failure.
14. Line 614, it should say Figure 5c instead of Figure 5d.
15. Lines 617 and 620, clarify if the correct reference is Figure 6c.

References

1. Wang, Lei, et al. "Bacteriophage–antibiotic combinations against ciprofloxacin/ceftriaxone-resistant Escherichia coli in vitro and in an experimental Galleria mellonella model." *International Journal of Antimicrobial Agents* 56.6 (2020): 106200.
2. Kumaran, Dilini, et al. "Does treatment order matter? Investigating the ability of bacteriophage to augment antibiotic activity against Staphylococcus aureus biofilms." *Frontiers in microbiology* 9 (2018): 127.
3. Chaudhry, Waqas Nasir, et al. "Synergy and order effects of antibiotics and phages in killing Pseudomonas aeruginosa biofilms." *PloS one* 12.1 (2017): e0168615.
4. Torres-Barceló, Clara, et al. "A window of opportunity to control the bacterial pathogen Pseudomonas aeruginosa combining antibiotics and phages." *PloS one* 9.9 (2014): e106628.
5. Leclerc, Quentin J., et al. "Growth-Dependent Predation and Generalized Transduction of Antimicrobial Resistance by Bacteriophage." *Msystems* (2022): e00135-22.
