# Supplementary material for: Modelling the synergistic effect of bacteriophage and antibiotics on bacteria: Killers and drivers of resistance evolution
Source: PLoS Comput Biol. 2022 Nov 30;18(11):e1010746. doi: 10.1371/journal.pcbi.1010746 (PMC9744316; doi:10.1371/journal.pcbi.1010746)
Supplement: Attachment — Submitted filename: Response to reviewers.docx [file pcbi.1010746.s009.docx]

Dear Editor,

Thank you for the opportunity to resubmit a revised version of our manuscript. We have implemented several changes in response to the reviewer comments which we believed have now strengthened the manuscript.

These changes notably include a restructuring of the Material and Methods and a clarification of our equations, as we previously relied too much on our original study which introduced the model (Leclerc et al., mSystems 2022), and therefore we did not initially include several explanations to support our equations in this manuscript.

We have also added multiple new sentences throughout the manuscript, as the reviewers recognized the interest of our work but suggested that the novelty should be clarified.

Finally, we added an additional paragraph in the Discussion to expand on the potential impact of bacterial resistance to phage on our results.

Please find below all the reviewer comments, and our point-by-point responses (in blue and italics). Note that the line numbers we refer to correspond to the line numbers in the revised manuscript with tracked changes.

Best regards,

Quentin Leclerc, on behalf of all co-authors

**Reviewer 1**

This an interesting study, investigating how timing of antibiotic and phage combination against Staph aureus affects bacterial clearance and multi-AMR evolution through horizontal gene transfer (generalised transduction) using deterministic numerical simulations. The results are intuitive: the application of antibiotics prior to phage reduces the killing power of phages but reduces the chance of multi-AMR, and vice versa if phages are applied first. The models are parameterised from in vitro data collected here and in previous work, but a wider range of parameter values are also explored. The findings are therefore likely to be relevant and reasonably generalisable.
*Thank you for your positive comments on our work.*

1. While interesting, the novelty of the work is perhaps marginal. The study is very similar to their previous (ref 23), but with timing of phage/antibiotic treatment altered. I would like the authors to be more upfront about this.

*Our previous study (ref 23, which has now become ref 28 in the revised text) introduced the phage-bacteria model, supported by in vitro experiments. However, the previous model did not include antibiotics. Instead, ref 23 explored multiple model structures in order to capture the interaction between phage and bacteria in the absence of antibiotics.*

*This difference is shown in the model diagram in this paper (Figure 1). Therefore, this work is a direct extension of our previous model, to introduce antibiotics and examine as you have correctly pointed out the effect of varying timings and concentrations of phage and antibiotics. We have emphasized this in Figure 1, and have clarified this in the text (lines 103, 162 & 637).*

2. Please mention lateral transduction as another mechanism of transduction. I saw a Penades paper cited, but could not find direct reference to this important phenomenon.

*We now mention lateral transduction as an important variation of specialised transduction in the Introduction (lines 70-73).*

3. Slightly odd to use a lysogenic phage as a phage therapy model, but the authors show that it is mainly lytic in these lab conditions. That said, bacteria typically evolve very rapid resistance lytic phage. Please discuss and speculate on impact on population and AMR evolution dynamics.

*In response to your suggestion and comments from other reviewers, we have now expanded the Discussion to include a paragraph on the potential impact of bacterial resistance to phage on our results (lines 674-694).*

**Reviewer 2**

The paper titled “Modelling the synergistic effect of bacteriophage and antibiotics on bacteria: killers and drivers of resistance evolution” explores how phage capable of generalized transduction impacts the efficacy of phage-antibiotics combination therapy. The authors claim that phage and antibiotics can work in combination to kill the bacterial population or to generate multidrug-resistant strains, the outcome will depend on the concentration and timing of phage and antibiotics application. The authors support their claims using a previously published mathematical model [5] which they extend by adding the pharmacodynamics of erythromycin and tetracycline, parameterized with new *in vitro* data from this study.

The claims of this manuscript contribute to the nascent field of phage-antibiotics combination therapy, proposing that generalized transduction and the timing and concentration of phage and antibiotics application shape the outcome of the combined treatment. However, these claims are not novel as there are previous experimental papers proposing similar ideas on the timing and concentration of phage-antibiotics application. For example, some studies have shown that pre-exposing biofilms to phage before adding the antibiotics produce the maximum anti-biofilm activity [1-3] while pre-exposing biofilms to antibiotics before adding phage can have antagonistic effects [2]. Similarly, the effects of timing and concentration of the combined treatment have been explored in planktonic bacteria, treating a bacterial population with phage prior to the addition of antibiotics resulted in the greatest bacterial density reduction [4].

I have outlined some major and minor comments below so the authors can consider them to improve their manuscript and to provide further support to their main claims.

*Thank you for pointing out these other studies, which we now reference as examples of other work looking at the joint effect of phage and antibiotics (lines 58-62). The novelty of our study which builds upon these previous experimental studies is (1) our use of a mathematical model, to explore a wide range of concentrations and timings, and hence better understand the conditions under which antibiotics and phage act in synergism or antagonism against bacteria.*

*While these previous studies concluded that introducing phage before antibiotics is better to clear bacteria, they only looked at a couple of timings (e.g. phage and antibiotics simultaneously, or with 24h delay between them), and a couple of concentrations (generally only one for phage, and one or two for antibiotics), which we now mention on line 62. They also often only measured the decrease in bacteria numbers after 24 or 48h, while we can observe the full dynamics over that period thanks to our model. We also note (2) that these studies mostly looked at biofilms, where the interaction between phage and antibiotics is different than here, since the aim is to use phage to disrupt the structure of biofilms and re-sensitise bacteria to antibiotics (as we now explain on lines 58-62). Finally, (3) only one of these studies looked at S. aureus, and most importantly, (4) to the best of our knowledge the impact of transduction of AMR genes has never been considered in such studies (as we state on line 67), making this a key novel aspect of our work.*

Major comments

1. Provide explanation/justification for some key aspects of the mathematical model. The main claims of the manuscript about the effects of timing and concentration and generalized transduction on the combination therapy outcome are supported by the mathematical model described in the manuscript. However, the model lacks some justification in some of its key aspects. For example, in equation 3 there is no explanation why the antibiotic effect (ε_i,j_) is coupled to the maximum bacterial growth rate (μmax_j_), as if an increase in the maximum bacterial growth rate increases the antibiotic effect. Then, equations 4-6 describe the population dynamics of the different bacterial strains but there is no explanation why their growth terms are coupled to the phage infection. For example, the growth term of bacteria resistant to erythromycin (B_E_) is described as $\mu_{E}(B_{E}- ((\varphi_{L}+{\varphi_{T})B_{E}))}$but there is no justification why phage infection negatively impacts replication of $B_{E}$. Then, is not clear how the parameters for bacterial growth and phage predations were obtained, whether they were calculated using the *in vitro* data from this manuscript or if they were obtained from [5]. Explaining key information that is missed from the components described above can be helpful and provide further support to the main results of the manuscript.

*Thank you for raising these points. Whilst all these elements were explained in our original study which introduced the model, we agree that we should have made them clearer here too. In response to your comments and suggestions from other reviewers, we have rephrased our Methods to better explain the model structure. In response to your specific examples, the antibiotic effect is coupled with bacterial growth to work with the bacterial growth parameter values from our previous studies (now explained on lines 191-193 and 326-328), and the negative impact of phage predation on bacterial growth results from our assumption that, over one time step, bacterial growth and phage infection are mutually exclusive events (lines 246-253).*

*For parameter values, please note that these are all listed in Table 1, alongside information on whether they were derived from this study, our original study which introduced the model (previously reference 23, now reference 28 in the revised text), or other studies (for the decay parameters).*

2. Proposing *in vitro* experiments to further support modeling outcomes. I think the manuscript could benefit from incorporating some simple experimental assays. The authors have the needed bacterial and phage strains for the proposed *in vitro* experiments. I propose to do some liquid co-cultures of the two bacterial strains resistant to single antibiotics and add phage and the antibiotics sequentially or simultaneously. The main idea is to quantify the effects of the combined treatment over time (via optical density or CFU counts) such that the experiment last ~24 to 48h. For example, in one experimental condition phage can be added first and later the antibiotics while in another condition antibiotics are added first and then the phage. In a third *in vitro* experiment phage and antibiotics are added simultaneously. Different concentrations of phage and antibiotics can be tested but I consider that using two is enough (high and low concentrations for both phage and antibiotics). The phage being capable of generalized transduction will produce the multidrug-resistant strains during the time frame of the suggested experiments (~24 to 48h); the multidrug-resistant strains could be selected when exposed to both antibiotics and then quantify. I consider that producing these experiments could provide further support to the modeling results. Even if the results from the *in vitro* experiments are different from the modeling outcomes, then the new information generated via experiments could be helpful to adjust the mathematical model.

*We fully agree that further in vitro work would be valuable, which is why experiments similar to what you have described have been started in our lab. However, setting up these experiments takes time, as we are interested in examining the phage-antibiotic-bacteria dynamics over a wide range of concentrations and timings, to build upon the hypotheses generated by our paper here. This was partly the aim of this modelling exercise, as highlighted on lines 99-107, to build upon existing in vitro data to explore multiple conditions and generate hypotheses to guide further in vitro work, instead of immediately conducting many experiments exploring multiple concentrations and timings.*

*However, at this stage we cannot add any further experimental results to this paper, as the full set of exploratory experiments take a substantial amount of time. Even for the conditions you have outlined, we currently do not have the corresponding data yet, and generating it is not feasible within the reviewing timeline for this paper (4 combinations of concentrations and 3 timings leads to 12 conditions, all taking place over 24h, and which would have to be repeated 3 times each, representing several months of full time work in the lab). We believe at this stage it will be much more valuable to present in the future the results from a wide range of timings and concentrations, rather than only generating a few combinations now, as this will better allow us to understand the differences between experiment results and model outcomes, if any.*

Minor comments

1. Line 57: add cites after the sentence, “Some previous studies showed that phage and antibiotics work synergistically to clear bacteria [add cites here]”.

*We have moved the citations here.*

2. Table 1, what are the correct units for the carrying capacity, *Nmax*, bacteria (CFU) or CFU/ml?

*We have corrected this to bacteria.mL^-1^.*

3. Table 1, what is the correct reference for bacterial growth and phage parameters, article 22 or 23?

*Thank you for spotting this mistake, the reference should have been [23], this has been corrected (note: this reference number has changed to 28 in this revised version).*

4. Table 1, what are the units for the half maximal effective concentration for the different antibiotics? mg/L?

*We have now added this information in the table.*

5. In line 296-297 the authors mentioned that the MIC of erythromycin for the sensitive strain was 0.25 mg/L and the same was found for tetracycline. But in line 320 authors mentioned that they simulated the addition of 1 mg/L of antibiotic which is 4 x MIC for susceptible strains and not 2 x MIC as stated. So clarify, if the MIC for susceptible strains is 0.25 mg/L or 0.5 mg/L.

*0.25 mg/L is the MIC measured via microbroth dilution, we have changed our text to clarify that 1 mg/L is therefore 4 x MIC.*

6. The error described above is found in different parts of the results section, for example, see line 346 from the caption of figure 3.

*We have corrected this throughout the manuscript.*

7. I think it would be clearer to show population dynamics plots for some of the combination therapy scenarios described in lines 382-392 than the current figure 5a.

*In response to your comment and suggestions by other reviewers, we have now reworked figures 5a and 5b to show these results as heatmaps for increased clarity. We believe that this, plus the explanations for the dynamics plots in 5c, are clear enough for the reader to understand how the timing and concentration of phage and antibiotics determines the outcome of the simulation.*

8. Line 476, maybe you could try to use parameters from the literature about antibiotic decay rates.

*That is what we have done by including these parameters in our partial rank correlation analysis (Figure 6) and showing the potential impact these decays may have in Figure S7.*

9. Lines 520-528, authors should point to the figures when mentioning the results described in those lines.

*We have now referenced the figures in this section.*

10. Lines 522-523, 2 mg/L of antibiotics is 4 or 8 x MIC?

*We have corrected this to 8 x MIC.*

11. Line 545, it should say Figure 6c instead of Figure 5c.

*Thank you for spotting this mistake which we have now corrected.*

12. Lines 551-553, maybe clarify if both sequential treatments can still lead to bacterial elimination.

*Thank you for this suggestion, we have now added this.*

13. Lines 581-582 it would be interesting to explore scenario where phage resistant bacteria are allowed to emerge. How does phage resistance affect the generation of multidrug-resistant bacteria? Or could phage resistance drive combined treatment to failure.

*We agree this would be interesting, and will be necessary in future work as some bacteria are known to be able to evolve phage resistance rapidly. Unfortunately, including this in our model was not possible here as it would have required a substantial redesign, adding 3 new compartments (one for each of the three bacterial strains, resistant to phage), and new data for parameterization which we could not generate as we did not see resistance appear in our lab mode within 24hl. We have now expanded this Discussion section to further discuss our opinion that phage resistance would lead to a higher risk of multidrug-resistant bacteria persistence (lines 674-694).*

14. Line 614, it should say Figure 5c instead of Figure 5d.

*Thank you for spotting this mistake, we have corrected this.*

15. Lines 617 and 620, clarify if the correct reference is Figure 6c.

*The reference should have been to Figure 6d, not 6c, thank you. We have moved this earlier in the sentence to clarify that we are referring to the observation that higher decay rates may lead to less bacteria remaining after 48h.*

References

1. Wang, Lei, et al. "Bacteriophage–antibiotic combinations against ciprofloxacin/ceftriaxone-resistant Escherichia coli in vitro and in an experimental Galleria mellonella model." *International Journal of Antimicrobial Agents* 56.6 (2020): 106200.
2. Kumaran, Dilini, et al. "Does treatment order matter? Investigating the ability of bacteriophage to augment antibiotic activity against Staphylococcus aureus biofilms." *Frontiers in microbiology* 9 (2018): 127.
3. Chaudhry, Waqas Nasir, et al. "Synergy and order effects of antibiotics and phages in killing Pseudomonas aeruginosa biofilms." *PloS one* 12.1 (2017): e0168615.
4. Torres-Barceló, Clara, et al. "A window of opportunity to control the bacterial pathogen Pseudomonas aeruginosa combining antibiotics and phages." *PloS one* 9.9 (2014): e106628.
5. Leclerc, Quentin J., et al. "Growth-Dependent Predation and Generalized Transduction of Antimicrobial Resistance by Bacteriophage." *Msystems* (2022): e00135-22.

**Reviewer 3**

‘Modelling the synergistic effects of bacteriophage and antibiotics on bacteria...' by Leclerc, Lindsay and Knight is a combined experimental and theoretical study into the impact of bacteriophage-mediated gene transfer (transduction) on the development of antimicrobial resistance in bacteria. The authors perform population dynamics experiment on S. aureus; they use these experiments to fit the parameters of a mathematical model, which they then use to explore a much wider parameter space. The main finding of this research is that, in their model, bacteriophage-mediated transduction can significantly increase the probability of multidrug-resistant bacterial mutants arising.

The conclusions of this article will be of interest to the wide community interested in combination phage/antibiotic therapy because they highlight the importance of generalised transduction in generating multidrug resistant bacteria. The general presentation of the work is very clear.

*Thank you for your comments as to the likely wide interest in this work.*

However, there are serious deficiencies in the presentation and clarity of the model that make it difficult for me to address the validity of these conclusions and I recommend this paper only be considered again after major revisions.

There are some serious errors and ambiguities in the model presentation. It is sometimes unclear to me what the model is and I am doubtful as to whether it represents what the authors want it to. Some of the criticisms below probably don't make sense because I have misunderstood what the authors are trying to do, but that is the point: I will need to see a revised manuscript to properly assess the model, as I cannot understand it yet.

*Thank you for highlighting this lack of clarity in model presentation. As mentioned above in response to another reviewer comment, we have extended a previous model in this manuscript, and many of the issues are linked to us not explaining this previous model in enough detail here. We have restructured the Methods to address this, including several new headings to separate the descriptions of the different model components.*

-Equations should be parts of sentences and have terminal punctuation. They should not be referred to before they are presented.

*We have now rephrased the equations throughout the manuscript, and changed the structure of the Methods to avoid referring to them before they are presented.*

-Variables like \mu{max} and \epsilon{max} look unwieldy and would be clearer with the max in superscript or some other notation.

*Thank you for this suggestion, we have now moved {max} to superscript for all parameters across the manuscript.*

-Eq. 3 should have the fraction written out fully, not y/x.

*We have corrected this in all equations.*

-Eq. 4 appears to have the (\psi_L+\psi_T)*B_E repeated; similarly for Eq. 5. Once this term is repeated with a prefactor of \mu_E (units of inverse hours) and once without, so this does not work dimensionally.

*Thank you for spotting this mistake resulting from a difference in how we phrased the equations compared to our original study which introduced the model. There should have been an ω parameter corresponding to one time-step (i.e. with units of hours) prefactoring the phi_L+phi_T term with the prefactor of mu_E on the right hand side of these equations. This was implicit in the sense that we consider that, over one time step, bacteria are either infected by phage or replicate (as now clarified on lines 246-253, in response to another reviewer comment), but we had not included that term to correct the dimensionality. This has now been fixed.*

-Asterisks are unnecessary to show multiplication and different parentheses {[()]} should be used to make nested expressions clearer, e.g., in Eq. 4.

*We have adjusted the parentheses throughout. We have now replaced asterisks with bullet points, in line with other PLoS Computational Biology articles (e.g.* [*https://doi.org/10.1371/journal.pcbi.1004782*](https://doi.org/10.1371/journal.pcbi.1004782)*).*

-In the eqs on p11 it's not obvious why certain terms are included or not, e.g., I would expect all phages to kill all bacteria, but only selected combinations seem to be represented. Likewise, it is not clear which terms correspond to lysis of bacteria by phage and what corresponds to transduction.

*P­_E_ and P_T_ are the generalised transducing phage (unable to replicate as they hold bacterial DNA, and not phage DNA, in their capsids), and P_L_ are the lytic phage. Therefore, only P_L_ can kill bacteria, which is represented in all equations, since P_E_ and P_T_ only carry a bacterial antibiotic resistance gene. As for P_E_ and P_T_, whilst they can infect all bacteria to transfer the gene they contain, this will not affect bacteria already carrying the corresponding gene (i.e. a B_E_ bacteria infected by P_E_ will still remain B_E_), which is why these interactions are not represented in the equations (they would cancel out).*

*We have now clarified this in lines 254-261.*

-Transduction requires that the transduced bacterium is not lysed by the phage and this lysis failure presumably also happens with bacteria that are not transduced. There should therefore be an overall lysis failure rate included.

*Since we are representing generalised transduction only, transducing phage cannot lyse bacteria. We have made this clearer in the text (line 169). However, nothing stops a bacteria from being lysed after it has moved to the B_ET_­ category, if it is infected by a lytic phage after transduction occurred. This is represented in the equations. Note that we only represent successful phage infections and lysis in the model, clarified on lines 213-215.*

-It is very helpful to have each term specified in words below these equations, as you do. We also need a clearer explanation of these equations, addressing the ambiguities raised above.

*We hope that these equations are now clearer thanks to the additional explanations we have provided in response to your comments.*

-\phi_{\theta} is defined over a page after it is first introduced and I don't think the definition is correct: \phi_{\theta} is presumably the probability that the bacterium is infected by the phage specified by \theta. If you want the probability of infection by at least one phage, that would be something like 1-\product_{\theta}(1-\phi_\theta).
*We did mean that this was the probability that a bacterium is infected by the phage of type P_theta, we have now rephrased this to clarify according to your phrasing (line 216).*

Assuming that the model is correct, the conclusions are interesting, and I appreciate how the authors have pinned down the many parameters in their model by the use of original experiments rather than relying on literature data. The sensitivity analysis is helpful, but we need to know rather more about how you fitted the parameters to the experiment. What fitting methods did you use and how did you (or did you) ensure that the fitted parameters were robust, i.e., can you adjust pairs or groups of parameters to give a similarly good fit to the model? This model has a large number of parameters and so I would want more information to be convinced that there is no qualitatively different set of parameters that will give as good a fit to the data. I understand that some of this work has already been done in ref. [23] but it needs to be summarized here too.

*Thank you for this suggestion. We have now added a brief explanation on how the model parameters were estimated in [23] on lines 313-317 (note this reference number has changed to 28 in this revised version). For the parameters related to antibiotic effect on bacterial growth, we estimated these using least squares methods, as described on line 318-329.*

I would also like the authors to explicitly state what the current work adds to reference [23] as this is not clear to me. Fig. 1 seems almost identical to a figure in ref. [23] and it would be helpful to acknowledge that here.
*This work extends the model from [23] (now reference 28 in text), with the addition of antibiotics. This is shown in Figure 1 by saying which components of the model are from the original one in [23], and which components are from the extended version here. We have a sentence in that figure caption to clarify this. We have also added a sentence on lines 162-163 to clarify this.*

The authors use various phrases to describe generalised transduction that seem to attribute some intentionality to the bacteriophage and bacteria, e.g., `accidentally' (l42, l65), `by mistake' (l67) and `a mispackaging and thus a biological error similar to a mutation' (l88). I object to this for two reasons: i) The whole of evolution is an `accident' in the same sense, so it's illogical to single out generalised transduction in this way and ii) These phrases would seem to imply that the transduction process gives no selective advantage for the bacteria or bacteriophage. As transduction generates drug-resistant bacteria, and hence more resilient hosts for the bacteriophage, this implicit claim at least needs some detailed, explicit justification. I suggest removing these and other similar phrases.
*Thank you for raising this interesting point. These choices of words arise from discussions with other reviewers on related work, with the aim to highlight that we currently have no strategy to prevent generalised transduction from happening. This is an important point with regards to phage therapy, as the risk of transduction is frequently dismissed in this setting with the claim that non-temperate phage will be used. Whilst this removes specialised transduction, generalised transduction remains, and cannot be prevented in the same way that mutations in the bacterial genome cannot be prevented, hence the importance of studying the consequences of this process. We believe our definition on line 98 is correct, as generalised transduction effectively occurs when the phage terminase incorrectly accepts bacterial DNA without the appropriate cos or pac sites. The process itself is therefore a mistake, which can sometimes benefit the phage and bacteria (as in the example you have provided), similar to mutations.*

*We have removed some of the phrases throughout the manuscript to avoid implying the intentionality you mentioned, and hope that our perspective is now clearer (lines 42, 69, 74).*

The data in Fig. 5 a-b is rather hard to read and there is no `red line' (l400) on these figures or at least it is not visible in my pdf. A clearer way to present this could be as a heat map, enabling the authors to systematically explore two control parameters simultaneously. The numbers on the axes of this figure, particularly the exponents, are too small and I recommend splitting this up into two figures.

*Thank you for this very helpful suggestion. We have now restructured Figure 5a and 5b as heatmaps. We have chosen not to split up the figure though, as 5c goes directly with 5b, and we believe it is more helpful for the readers to see 5a and 5b together.*
