# Supplementary material for: Modelling the synergistic effect of bacteriophage and antibiotics on bacteria: Killers and drivers of resistance evolution
Source: PLoS Comput Biol. 2022 Nov 30;18(11):e1010746. doi: 10.1371/journal.pcbi.1010746 (PMC9744316; doi:10.1371/journal.pcbi.1010746)
Supplement: Attachment — Submitted filename: Response to reviewers.docx [file pcbi.1010746.s010.docx]

Dear Editor,

Thank you for the opportunity to resubmit a revised version of our manuscript. In light of the comments from Reviewer 3 about our model, and further discussions with colleagues, we have revised our equations describing phage-bacteria interactions to improve the mathematical and biological clarity of the model.

Briefly, our original equations effectively imposed a maximum value on the rate at which phage and bacteria interacted. However, our implementation of this restriction led to some mathematical inconsistencies, as highlighted by Reviewer 3. To overcome this, we have improved our equations to restrict phage predation via a Hill function. This approach was originally introduced in Roach et al 2017 (reference 43), and reflects the same biological process and decision making behind our original model.

We have refitted this new model version, which is exactly the same apart from the function for phage-bacteria interaction and requires only one additional parameter (P50, the concentration of phage at which predation is half-saturated). The resulting parameter values do not substantially differ from the original ones. As this is in effect a different mathematical expression of the same processes, this improved equation does not substantially change our results and conclusions.

Please find below the reviewer comments, and our responses (in blue and italics). Note that the line numbers we refer to correspond to the line numbers in the revised manuscript with tracked changes.

Best regards,

Quentin Leclerc, on behalf of all co-authors

**Reviewer 1**

You have done a very good job responding to reviewer comments. Thank you for producing an interesting paper.

*Many thanks for your positive assessment of our manuscript.*

**Reviewer 3**

The authors have made considerable efforts to improve the article, e.g., Fig. 5 is now much clearer, and they have addressed most of my concerns. However, although I now have a clearer picture of the model, I don't believe it is a coherent model, either in the implementation here, or in the authors' previous paper, ref. 28, doi:10.1128/msystems.00135-22. I am not optimistic that the authors will be able to resolve these issues without further major revisions so I would recommend that the paper be rejected.

Specifically, the authors mix discrete and continuous equations in an ad hoc fashion without sufficient justification. If Eq. 8 is supposed to be a differential equation rather than a difference equation it shouldn't explicitly reference the `time-step', which is 1/omega. If it's a difference equation, it should be displayed as such. Also, I'm puzzled as to what the authors mean by a `time-step' here at all. It is clear from the figures that the numerical integration is performed more frequently than once per hour, whereas omega=1/hour.

I agree with the authors about the problem: if the `timestep' is very long then the number of bacteria can become negative. There are two ways to deal with this: one is to use a much smaller time step and then, if necessary, deal with very small bacterial numbers by imposing an arbitrary cut off (e.g., if the total bacterial number in the system drops below one, that number could be set to zero). The other way of dealing with this, which the authors attempt, is to add correction terms to give the correct probabilities of infection and growth within much longer time steps. However, the authors do this in an ad hoc fashion, by just adding the term containing omega to Eq. 8. This step is not justified and I am not convinced that they have kept all terms to lowest order in omega, or that higher order terms (omega^2 etc.) are not needed with such a long time step.

In any future version, I would expect the authors to start from an appropriate differential equation model and derive their difference equation model (if that is what it is) formally, justifying all steps mathematically and making sure that the meaning of all terms (such as the `timestep') is clearly defined, and it is clear how they are related, e.g., how is the delay between infection and lysis related to the `timestep'?

*Thank you for your positive comment on this improved version. We recognise that the original formulation of the so called “frequency-dependent” interaction in this work was ambiguous, and led to some mathematical inconsistencies.*

*Briefly, the justification for this interaction came from our observation that high concentrations of phage (P) did not lead to a sharp decline of bacteria (B). The most commonly used method to represent phage-bacteria interaction is linear (B*P*β), and consistently predicts this sharp decline which we did not observe. To overcome this, we effectively imposed a restriction on the P*β multiplier that was applied to B, but we recognise that this led to some mathematical inconsistencies as you have highlighted.*

*To overcome this issue whilst maintaining this restriction on phage-bacteria interaction, we have replaced our original equations by a Hill function. This approach was originally introduced in Roach et al 2017 (reference 43), as it better captured phage-bacteria dynamics than the classical linear equation (B*P*β). For any phage theta, we now express the number of phage F(P_θ_) (θ ∈ {L, E, T}, respectfully lytic, ery-transducing, and tet-transducing phage) infecting any bacteria as*

$$F\left( P_{\theta} \right)=P_{\theta}\cdot\frac{\beta}{1+\frac{P_{\theta}}{P50}}$$

*With β being the maximum interaction, and P50 the phage concentration at which the interaction is half-saturated. The Methods have been updated to reflect this change (lines 207-280).*

*We have refitted the model to our original in-vitro data, as it contains an additional parameter (P50). This has led to a slight increase in the transduction probability (from 1.0 x 10^-8^ to 1.19 x 10^-8^), which we found to be in better agreement with our data. This leads to a slightly increased rate of multidrug-resistant bacteria generation, visible in the updated Figures 3-6.*

*Otherwise, updated parameter values do not differ substantially from the previous ones, as can be seen in Table 1. Furthermore, as this is in effect a different mathematical expression of the same processes captured by our original model, this improved equation does not substantially change our conclusions on the potential for phage and antibiotics to act both as synergistic killers of bacteria, or drivers of AMR evolution, depending on timing and concentration.*

*We recognise that this mathematical inconsistency was also present in our previous paper (ref 28), which is why we have submitted an Author Correction for this paper, which has been accepted by the journal and will be available shortly.*

Also, when describing Eq. 3 it would be clearer to describe epsilon_{i,j} as the antibiotic-induced death rate (or similar) rather than just the `effect' of the antibiotic. I struggled for a bit because I assumed that `effect' should be a factor that is multiplicative on the growth rate.

*Thank you for this suggestion, which we have implemented on lines 180-185, 297-309, 369-371, and in Table 1.*
